# Experimental pasta as an innovative approach to cholesterol reduction in patients with metabolic syndrome, with and without major psychiatric disorders: A randomized controlled trial supported by *in vitro* validation

**DOI:** 10.1192/j.eurpsy.2025.10057

**Published:** 2025-07-07

**Authors:** Enrico D’Ambrosio, Maria Favia, Marcello Greco Miani, Francesco Pappagallo, Laura De Mastro, Antonia Ianniello, Silvia Saltarelli, Maria Fiore, Giulia Napoletano, Rita Masellis, Agostino Di Ciaula, Mohamad Khalil, Elisa Lanza, Antonino Noto, Felice Ungaro, Gianluca Kikidis, Giulio Pergola, Piero Portincasa, Alessandro Bertolino, Precision Healthcare and Lifestyle Working Group, Antonio Rampino

**Affiliations:** 1 Department of Translational Biomedicine and Neuroscience (DiBraiN), University of Bari “Aldo Moro”, Bari, Italy; 2 Azienda Ospedaliero Universitaria Consorziale Policlinico, Bari, Italy; 3 Casillo Group, Corato, Italy; 4 Consis, Bari, Italy; 5 Department of Precision and Regenerative Medicine and Ionian Area (DiMePRe-J), University of Bari “Aldo Moro”, Bari, Italy; 6 Health Marketplace and Puglia Life Science Foundation, Bari, Italy; 7 Lieber Institute for Brain Development, Johns Hopkins Medical Campus, Baltimore, MD, USA; 8 Department of Psychiatry and Behavioral Sciences, Johns Hopkins School of Medicine, Baltimore, MD, USA

**Keywords:** antipsychotics, hypercholesterolemia, major psychiatric disorders, metabolic syndrome, psychopharmacological treatment

## Abstract

**Background:**

Elevated non-high-density lipoprotein cholesterol (non-HDL-C) is a significant risk factor for atherosclerotic cardiovascular diseases, particularly in individuals with metabolic syndrome (MetS) and major psychiatric disorders (MPDs), who may experience metabolic side effects of psychopharmacological treatments. We evaluated the cholesterol-lowering effects of an experimental pasta characterized by a high content of phytosterols, arabinoxylans, polyunsaturated and monounsaturated fatty acids, and vitamin E in individuals with MetS, with and without MPDs.

**Methods:**

In a double-blind, randomized trial, 298 participants with MetS were assigned to consume either experimental or conventional pasta for 3 months. Non-HDL-C levels were measured at baseline and follow-up. A polygenic risk score for hypercholesterolemia (TC-PRS) was calculated to assess any genetic influence on the intervention’s efficacy. The cholesterol-lowering effect of the experimental pasta was also tested *in vitro* by exposing human hepatocarcinoma cells, which developed lipid storage alterations due to olanzapine (OLZ) exposure, to an extract of the flour mixture used to prepare the experimental pasta.

**Results:**

The participants who consumed the experimental pasta exhibited a significantly greater reduction in serum non-HDL-C levels compared to the control group (*p* = 0.001). No significant interaction between pasta variety and the TC-PRS on non-HDL-C changes was found. The extract from the experimental flour mixture significantly reduced both the number and size of lipid droplets in HepG2 cells treated with OLZ.

**Conclusions:**

These results indicate that a low-impact lifestyle intervention may offer a practical strategy for improving the cholesterol profile and mitigating cardiovascular risk in patients with MetS, with and without an MPD.

## Introduction

Hypercholesterolemia is a significant risk factor for atherosclerotic cardiovascular diseases [1–4], which, in turn, are responsible for approximately one-third of ischemic heart disease and one-fifth of global cerebrovascular disease [5–7] in the general population. In particular, non-high-density lipoprotein cholesterol (non-HDL-C), calculated by subtracting HDL cholesterol (HDL-C) from total cholesterol (TC), is a predictor of cardiovascular disease mortality [8, 9] and is commonly used in clinical guidelines as an indicator of cardiovascular risk [7, 9–11].

Importantly, studies report that unhealthy nutritional habits are a major contributor to the risk for hypercholesterolemia in the general population and specifically in patients with major psychiatric disorders (MPD). Psychopharmacological treatments, including second-generation antipsychotics (SGA), antidepressants, and mood stabilizers, may induce metabolic alterations *per se* [12–19], increasing the risk for elevated non-HDL-C levels and metabolic syndrome (MetS) in patients with MPD, while serum non-HDL-C increase in these patients is also associated with poor dietary habits.

However, genetic factors may significantly contribute to the risk for hypercholesterolemia, thereby interacting with nutritional and other environmental variables on the pathogenesis of this condition. In fact, in addition to familial hypercholesterolemia [20], evidence supports a polygenic contribution to nonfamilial hypercholesterolemia [21]. In this regard, recent large-scale meta-analyses of genome-wide association studies (GWASs) have demonstrated that numerous common genetic variants are associated with hypercholesterolemia [21–29].

Importantly, serum cholesterol level is only an indirect measure of cholesterol dysmetabolism in the organism, which includes abnormal lipid accumulation in different organs, such as the liver, the muscle, and the perivisceral adipose tissue. In this regard, a strategy to investigate cholesterol metabolism at the organ level is the quantification and qualitative analysis of lipid droplets, which are regularly stored in specific cell types and the hepatocytes in particular. In fact, hepatic lipid droplets serve as intracellular organelles for lipid storage, and their number and size may reflect a hepatic cholesterol overload [30–34] along with a general lipid accumulation. Within this perspective, the human hepatocellular carcinoma (HepG2) cell line has been extensively utilized as an *in vitro* model to study lipid droplet formation and cholesterol metabolism [35, 36]. Interestingly, while HepG2 cells can also be used to study the effects of nutraceutical substances on lipid metabolism due to their ability to absorb, synthesize, and store lipids [37–39], they represent an ideal model for studying the effects of drugs on lipid metabolism [40, 41]. For instance, HepG2 cells have been used to study the impact of SGAs on lipid metabolism, and studies using this approach have shown, for example, that an increase in intracellular lipid droplets can follow HepG2 cell exposure to olanzapine (OLZ) [42], an SGA with a high potential to induce hypercholesterolemia [43].

Among nutrients with the highest impact on systemic cholesterol balance, carbohydrates, including those contained in flour derivatives, such as bread and pasta, play an important role. In particular, pasta, one of the most consumed foods worldwide [44, 45], is a key component of the Mediterranean diet, whose importance in cholesterol balance has broadly been documented [46, 47]. Additionally, studies have indicated that pasta nutrient composition can be modified in order to give this food a pro-metabolic effect on the cholesterol profile [48, 49]. In this regard, here we tested the effect of a pasta specifically developed to have a nutritional profile aimed at reducing serum cholesterol levels compared to conventional pasta. This experimental pasta was made from a blend of semolina (SEMOL) and wheat germ oil (in microencapsulated form) with a high content of phytosterols, a class of fat-soluble compounds known to reduce plasma cholesterol levels [50, 51], especially by decreasing cholesterol gut absorption [52]. Specifically, given their structural similarity to cholesterol, phytosterols compete with both dietary and biliary cholesterol for incorporation in mixed micelles and consequent absorption into enterocytes via the Niemann-Pick C1-like 1 transporter [52–55]. Notably, the experimental pasta was also significantly richer in arabinoxylan content compared with conventional pasta (4.4 g/100 g and 1.8 g/100 g, respectively). This is a particularly relevant aspect, as robust evidence suggests that arabinoxylans have hypocholesterolemic effects [56–59]. In particular, studies suggest these compounds may induce a reduction of cholesterol synthesis by inhibiting HMG-CoA reductase activity and an increase in its decomposition into bile acids by modulating hepatic CYP7A1 [60–62]. Furthermore, they downregulate cholesterol absorption at the gut level [63]. Another factor contributing to a potential hypocholesterolemic effect of experimental pasta was this food’s higher content of polyunsaturated fatty acids (PUFAs) and monounsaturated fatty acids (MUFAs) compared to conventional durum wheat pasta. In this regard, even though with some exceptions [64], extensive evidence demonstrates that PUFAs exert a significant serum cholesterol-lowering effect [65, 66], while previous studies have highlighted cholesterol reduction associated with high MUFA diets [67–71]. Additionally, the experimental pasta used in the present study was significantly richer in vitamin E compared to conventional pasta, and previous reports have suggested that vitamin E complex compounds have serum cholesterol-lowering effects in both clinical and preclinical paradigms [72–76].

To assess the beneficial effects of experimental pasta on the non-HDL-C profile of patients with MetS, here we adopted a multistep approach in which (1) we performed a double-blind randomized clinical trial comparing the impact of experimental and conventional pasta on serum non-HDL-C level modification over a period of 3 months; (2) we investigated whether pasta variety interacted with genetic risk for cholesterol increase on serum non-HDL-C variation; and (3) we tested the lipid-lowering effect of an extract from the flour mixture used to prepare the experimental pasta in an *in vitro* cell model.

To generalize the findings, we included subjects from two distinct populations, both characterized by the presence of the MetS phenotype: participants recruited from the general population and individuals with an MPD who were undergoing stable psychopharmacological treatment. We hypothesized that incorporating experimental pasta into participants’ diets would significantly reduce their serum non-HDL-C levels compared to individuals consuming conventional (control) pasta in a 3-month time window. Explorative analyses were performed to assess the effect of the experimental pasta on other lipid parameters, such as TC, low-density lipoprotein cholesterol (LDL-C), HDL-C, and triglycerides.

Furthermore, for each individual, we calculated a polygenic risk score for serum TC increase (TC-PRS) and assessed the interaction between this score and the variety of pasta consumed on serum non-HDL-C change within the same time window. Additionally, we explored the impact of a bioactive extract derived from the flour mixture used to prepare the experimental pasta on a cell model that reproduces an antipsychotic-induced lipid dysmetabolism in the hepatocyte.

## Methods

### Clinical trial

#### Study design

The longitudinal parallel arm double-blind randomized trial was conducted at the University of Bari “Aldo Moro,” Italy. The study was performed in accordance with the International Declaration of Helsinki and the International Council for Harmonization of Technical Requirements for Pharmaceuticals for Human Use Good Clinical Practice guidelines, and the study protocol was approved by the local ethical committee (*Comitato Etico Indipendente, Azienda Ospedaliero-Universitaria Consorziale Policlinico, Bari*).

#### Subjects

Participants were consecutively recruited from two outpatient units, an internal medicine unit dedicated to outpatients with MetS (INT MED sample) and a psychiatry unit dedicated to outpatients with MPDs (including schizophrenia spectrum disorders, bipolar disorder, major depressive disorder, and obsessive-compulsive disorder) who had developed a MetS while assuming psychotropic medications (antipsychotics, antidepressants, mood stabilizers, and benzodiazepines in mono- or polytherapy) at a stable dose for at least 1 month (MPD sample). Diagnoses were formulated by INT MED and psychiatry specialists. Detailed inclusion and exclusion criteria are reported in the Supplementary Materials. All participants provided written informed consent before entering the study.

#### Pasta composition

Experimental pasta was developed by Casillo Next Gen Food srl (Corato, BA, Italy) according to a pending patent (IPN WO 2023/242753 A1) using a blend of 67% SEMOL, 27% defatted wheat germ, and 6% microencapsulated wheat germ oil along with a high-protein (15.8 g/100 g), fiber (total fiber 9.5 g/100 g of product), and arabinoxylan (4.4 g/100 g of product) content [77]. Moreover, the experimental mixture adopted was rich in PUFAs, MUFAs, vitamin E, and total phenols.

Conventional pasta, used as a placebo, was SEMOL pasta (as defined by the decree of the President of the Italian Republic No. 187 – February 9, 2001) made from conventionally processed SEMOL, with added food coloring to make it indistinguishable from the experimental pasta.

#### Randomization and masking

In each group, subjects were randomized (1:1) using the online tool *Research Randomizer* (https://www.randomizer.org/) to receive experimental or placebo pasta. Experimental and placebo pasta were identical in appearance and were delivered to patients by researchers in a double-blind manner. The experimental allocation of pasta (experimental or placebo) was kept confidential from the participants, investigators, and the sponsor of the study throughout the entire treatment and monitoring phase until the database was secured.

#### Procedures

For the whole study duration, that is, 3 months, each participant was asked to maintain the same lifestyle and dietary regimen as before enrollment, except substituting their usual pasta with the pasta provided by the researcher. This decision was made to prevent other potentially cholesterol-impacting confounding variables from influencing the trial results.

Importantly, no instructions were given regarding the quantity of pasta to consume, as the participants were asked to determine such a quantity based on their ordinary dietary habits. We chose this approach in order to ensure that the nutritional intervention we introduced with the current trial aligned as closely as possible with the participants’ usual dietary patterns, thereby minimizing disruption to their routine and enhancing the ecological validity of the study. To establish daily pasta consumption (and consequently the quantity of pasta to be delivered by researchers), a questionnaire was administered at the time of study entry, T0. Therefore, the pasta quantity provided during the trial matched each participant’s habitual consumption at baseline. As expected, there were no statistically significant differences (*p* = 0.261) in the amount of pasta consumed between the groups in the experimental pasta arm over the 3-month trial (mean = 6.832 kg, SD = 0.294 kg) and the placebo pasta arm (mean = 6.365 kg, SD = 0.291 kg).

At T0, subjects underwent venous blood sampling, medical history collection, demographic and anthropometric data collection, administration of a standardized questionnaire to assess adherence to the Mediterranean diet [78, 79], administration of the Structured Clinical Interview for DSM Disorders (SCID), a semi-structured interview for psychiatric diagnosis according to the Diagnostic and Statistical Manual of Mental Disorders, Fifth Edition (DSM-5) diagnostic criteria [80], and administration of the Eating Attitude Test (EAT26) [81] to rule out the presence of any diagnosis of eating disorder. Physical activity was assessed with the International Physical Activity Questionnaire (IPAQ), a validated instrument that quantifies self-reported physical activity as a combined total score expressed in MET-minutes per week [82, 83]. After 3 months of consuming pasta provided in the study (T1), subjects underwent a new assessment, including anthropometric measurement, a second venous blood sample collection, and re-administration of the Mediterranean diet adherence questionnaire and IPAQ. For TC, laboratory results were unavailable for one subject at baseline (T0) and 4 subjects at follow-up (T1). For HDL-C, results were unavailable for two subjects at baseline and four subjects at follow-up. For LDL-C, results were unavailable for 19 subjects at baseline and 5 subjects at follow-up. For triglycerides, results were unavailable for four subjects at baseline and one subject at follow-up.

#### Genotyping and PRS calculation

All individuals in the study were whole-genome genotyped, and genomic variants associated with total blood cholesterol increase according to the latest and largest, to our knowledge, GWAS study [28] were used for hypercholesterolemia (TC increase) PRS (TC-PRS) computation (detailed methods reported in the Supplementary Materials).

#### Statistical analysis

Descriptive baseline characteristics are presented in Table 1. Baseline comparability of the two study arms was assessed with *χ*^2^ tests for categorical variables and Mann–Whitney *U* tests for continuous variables.

**Table 1.** Samples’ baseline characteristics (randomized subjects)

	Total sample	MPD	INT MED
*N*	298	97	201
Gender (M/F)	141/157	49/48	92/109
Age (years) mean (SD)	53.497 (14.800)	44.814 (13.114)	57.687 (13.731)
BMI (kg/m2) mean (SD)	30.713 (5.586)	32.972 (6.346)	29.622 (4.830)
Abdominal circumference (cm) mean (SD)	105.435 (13.294)	109.665 (14.082)	103.393 (12.426)
Systolic BP (mmHg) mean (SD)	124.610 (12.393)	123.188 (10.771)	125.296 (13.074)
Diastolic BP (mmHg) mean (SD)	77.959 (8.830)	77.927 (9.283)	77.975 (8.627)
Non-HDL cholesterol (mg/dl) mean (SD)	136.969 (41.985)	142.566 (44.731)	134.231 (40.417)
Total cholesterol (mg/dl) mean (SD)	184.595 (40.928)	186.832 (44.537)	183.537 (39.179)
LDL cholesterol (mg/dl) mean (SD)	114.468 (40.618)	125.478 (43.382)	109.082 (38.171)
HDL cholesterol (mg/dl) mean (SD)	47.931 (14.514)	45.679 (13.039)	49.000 (15.078)
Triglycerides (mg/dl) mean (SD)	139.671 (90.683)	160.237 (134.840)	130.060 (57.848)

*Note*: The table reports the demographics of samples recruited in the study, along with anthropometric and blood measures related to MetS. MPD, major psychiatric disorders; INT MED, internal medicine unit dedicated to outpatients with MetS.

Linear mixed-effects models were used to analyze changes in non-HDL-C (primary outcome), accounting for repeated measures within participants by including a random intercept for each subject. Fixed effects were pasta arm (experimental vs. placebo pasta), time (baseline [T0] and 3-month follow-up [T1]), and their interaction (time-by-pasta), adjusting for age, gender, and patient category (MPD vs. INT MED). The primary effect of interest was the time-by-pasta interaction. Analyses were conducted according to the modified intention-to-treat (mITT) principle and repeated to confirm the results using the ITT approach, with the missing data imputed using the baseline-observation-carried-forward method. In addition, exploratory subgroup analyses were conducted to evaluate the treatment effect within the MPD and INT MED patient categories.

Exploratory analyses separately evaluated the potential influence of additional covariates, including physical activity (MET-minutes per week from IPAQ), statin use, antipsychotic medication use, and Mediterranean diet adherence score. Further exploratory analyses were conducted using TC, LDL-C, HDL-C, and triglycerides instead of non-HDL-C. Finally, TC-PRS was tested as a potential moderator of the treatment effect through a three-way interaction (time-by-pasta-by-TC-PRS). All tests were two-tailed, with a significance threshold of *α* = 0.05. No correction for multiple testing was applied, given that the study was designed with a single primary endpoint (non-HDL-C) and all secondary analyses were considered exploratory. Analyses were performed with R v4.5.0 using the lme4 [84] and lmerTest [85] packages.

### In vitro HepG2 experiment

#### Cell culture and treatments

HepG2 human hepatocarcinoma cells were left to attach for 24 h and were then exposed for the first 72 h to 10 μM OLZ and for the next 72 h either to the extract derived from the experimental mixture (NUTR) or to SEMOL (2 mg). Detailed methods are reported in the Supplementary Materials.

#### Lipid droplet staining and imaging analysis

HepG2 cells were stained with 0.5 Oil Red O. ImageJ software and the methodological protocol provided by Adomshick et al. [86] were used to analyze the images (Figure 3A) and quantify lipid particles, as well as the total area occupied by them in the brightfield microscopy images. Details are reported in the Supplementary Materials.

#### Statistical analyses

An unpaired Student’s *t*-test was used to compare HepG2 lipid droplet number and size between different cell exposure conditions. In particular, cell exposure conditions were “untreated,” that is, exposed to vehicle (UNTR), “exposed to OLZ” (OLZ), or “exposed to experimental mixture extract” (NUTR) and SEMOL. Inter-condition comparisons were made between each OLZ or NUTR and UNTR cells, as well as between cells treated with OLZ and cells treated with the combination of OLZ and NUTR (OLZ vs. OLZ + NUTR). Statistically significant differences were defined as *p*-values < 0.05. Experiments comparing the effects of cell exposure to OLZ + SEMOL versus OLZ are described separately in the Supplementary Materials.

## Results

### Clinical trial

**Figure 1.**
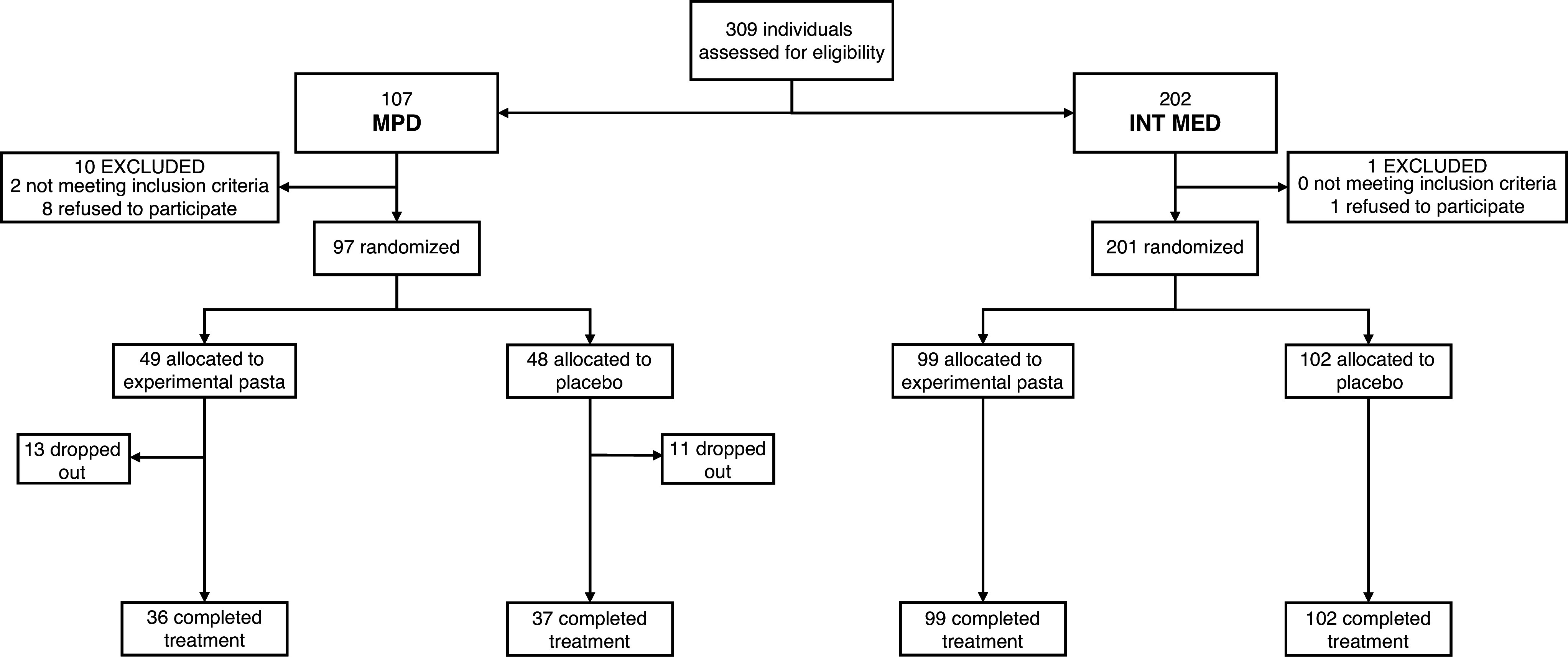
Trial flowchart.

Participants were recruited between March 11, 2023, and October 28, 2023; the last participant completed the trial on January 29, 2024. A total of 309 participants were screened for eligibility, of whom 298 were randomly assigned to receive either experimental (*n* = 148) or placebo (*n* = 150) pasta. Ninety-seven of these patients were recruited from the psychiatry (MPDs) and 201 from the INT MED outpatient unit. After the randomization, 24 patients recruited in the psychiatry unit (experimental pasta arm, *n* = 13; placebo pasta arm, *n* = 11) dropped out and returned the pasta provided. Overall, 274 participants completed the trial (Figure 1). Demographics and baseline data are reported in Table 1.

Baseline demographic, clinical, and lifestyle characteristics were balanced between experimental and placebo arms; specifically, no statistically significant differences emerged regarding age, gender, body mass index, abdominal circumference, systolic and diastolic blood pressure, non-HDL-C, TC, LDL-C, HDL-C, triglycerides, total physical activity (MET-minutes per week), or Mediterranean diet adherence score (all *p* > 0.1).

**Figure 2.**
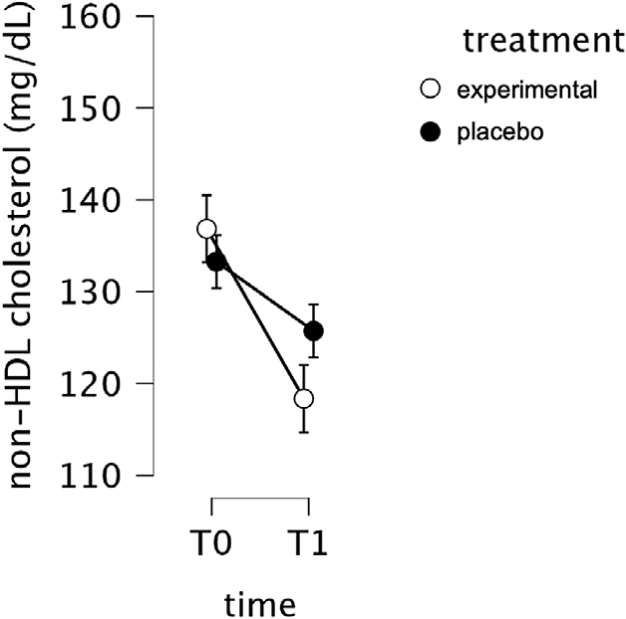
Change of serum non-HDL cholesterol levels from T0 to T1 for experimental and placebo pasta.

At the 3-month follow-up, non-HDL-C significantly decreased more with experimental pasta than with placebo (mITT analysis, time-by-pasta interaction: *p* = 0.001) (Figure 2 and Supplementary Table 1A). The mean change in non-HDL-C at week 12 was −18.488 mg/dl (SD = 28.650) for patients who had consumed experimental pasta and −7.550 mg/dl (SD = 23.626) for those who had used placebo pasta (Table 2). The ITT analysis, including all randomized subjects, also showed a significant time-by-pasta (experimental or placebo) interaction (*p* = 0.001).

**Table 2.** Measures at baseline (T0) and follow-up (T1) for subjects who completed the trial

	Placebo			Experimental		
	T0 mean (SD)	T1 mean (SD)	Mean difference (SD)	T0 mean (SD)	T1 mean (SD)	Mean difference (SD)
Non-HDL cholesterol (mg/dl)	133.275 (38.749)	125.725 (42.036)	−7.550 (23.626)	136.846 (41.100)	118.358 (34.901)	−18.488 (28.650)
Total cholesterol (mg/dl)	183.993 (39.346)	178.642 (40.222)	−5.350 (18.860)	183.432 (39.495)	170.136 (33.619)	−13.295 (27.053)
LDL cholesterol (mg/dl)	111.747 (38.101)	105.275 (34.923)	−6.683 (25.987)	111.558 (36.961)	105.075 (33.219)	−6.307 (23.895)
HDL cholesterol (mg/dl)	49.603 (15.251)	50.846 (16.210)	1.313 (12.827)	46.670 (13.967)	50.303 (13.741)	4.238 (11.962)
Triglycerides (mg/dl)	133.519 (67.104)	131.089 (69.775)	−2.430 (47.707)	137.223 (61.033)	128.431 (61.607)	−8.792 (57.933)
BMI (kg/m^2^)	30.544 (5.407)	30.291 (5.363)	−0.253 (1.306)	30.287 (5.453)	30.051 (5.686)	−0.237 (1.23)
Abdominal circumference (cm)	105.61 (13.705)	105.371 (13.962)	−0.239 (6.877)	104.817 (13.231)	105.02 (14.004)	0.202 (6.319)
Systolic BP (mmHg)	125.518 (12.169)	126.302 (12.583)	0.784 (10.147)	124.068 (13.216)	124.424 (12.923)	0.356 (9.361)
Diastolic BP (mmHg)	77.777 (8.325)	80.36 (9.294)	2.583 (9.858)	77.909 (9.412)	78.674 (9.568)	0.765 (9.985)

*Note*: Table reports the measures at the two study timepoints (T0 and T1), along with the related mean differences in the two arms of the trial.

No statistically significant changes were observed from baseline to follow-up in Mediterranean diet adherence scores (*p* = 0.231) or total physical activity (*p* = 0.702); pharmacological treatments remained stable throughout the trial as per protocol. The time-by-pasta interaction remained consistent when adjusted for physical activity (*p* = 0.001), statin therapy (*p* = 0.001), antipsychotic medication (*p* = 0.001), and Mediterranean diet adherence (*p* = 0.001), indicating no confounding effects by these covariates (Supplementary Table 1A).

Exploratory analyses on other lipid parameters revealed a statistically significant interaction between time and pasta (experimental or placebo) on TC (*p* = 0.005) (Supplementary Table 1B). The mean change in TC at week 12 was −13.295 mg/dl (SD = 27.053) for patients who had consumed experimental pasta and −5.350 mg/dl (SD = 18.860) for those who had used placebo pasta (Table 2). The ITT analysis, including all randomized subjects, also showed a significant interaction between time and pasta variety (*p* = 0.006). The results of the analyses exploring the effects of pasta on LDL-C, HDL-C, and triglycerides were not statistically significant (all *p* > 0.1) (Supplementary Table 1B).

TC-PRS was not significantly correlated with baseline non-HDL-C (*p* = 0.653). Furthermore, the interaction between time, pasta variety, and TC-PRS on non-HDL-C was not statistically significant (*p* = 0.186) (Supplementary Table 1A).

In subgroup analyses, experimental pasta significantly reduced non-HDL-C compared to placebo in INT MED participants (time-by-pasta interaction: *p* < 0.001), whereas no significant difference emerged in the MPD subgroup (time-by-pasta interaction: *p* = 0.772) (Supplementary Table 1C). In the MPD subgroup, mean non-HDL-C reductions were −16.682 mg/dl (SD = 36.158) for patients who had consumed experimental pasta and −14.257 mg/dl (SD = 33.642) for those who had used placebo pasta; in the INT MED group, reductions were −19.172 mg/dl (SD = 25.448) for patients in the experimental pasta arm and −5.104 mg/dl (SD = 18.335) for patients in the placebo arm.

### In vitro HepG2 experiment

Cell viability assays were performed to ensure treatments to which HepG2 cells were exposed were not cytotoxic. No detrimental effects on cell viability after exposure to 10 μM of OLZ and 2 mg of the NUTR or SEMOL extracts were observed (data not shown).

**Figure 3.**
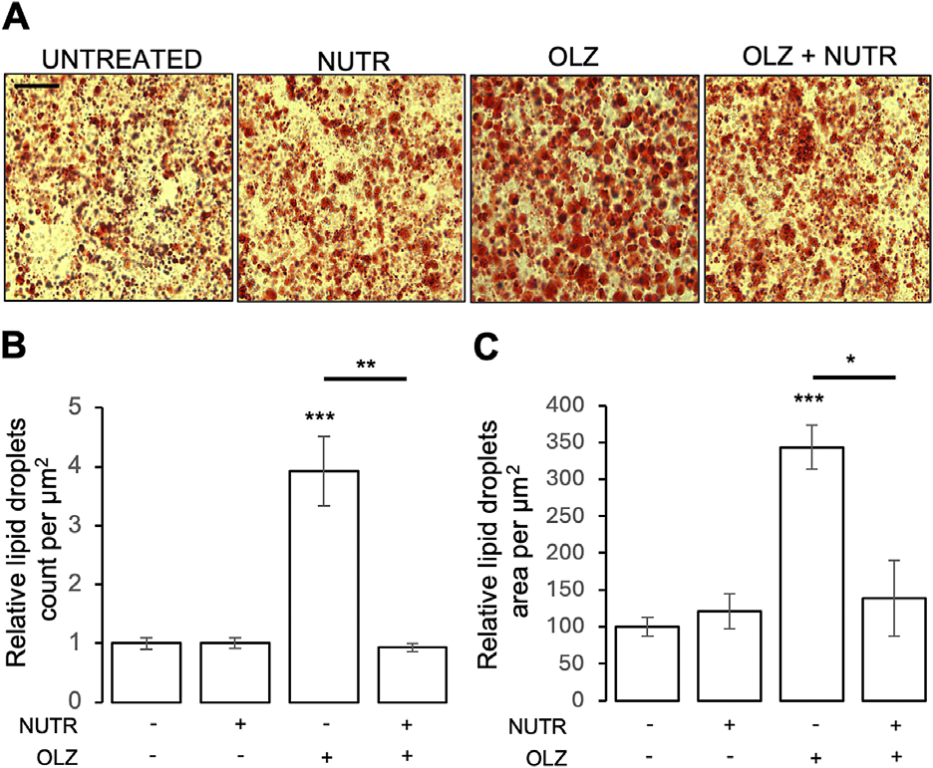
Effect of nutraceutical and olanzapine treatments on lipid droplet formation in HepG2 cells. (A) Representative images showing lipid droplets in HepG2 cells under different conditions, stained by Oil Red O. The images were captured using brightfield fluorescence microscopy at 20× magnification. The conditions are as follows: NUTR – treatment with a 2 mg experimental flour (nutraceutical) extract for 72 h; OLZ – treatment with 10 μM olanzapine for 72 h; OLZ + NUTR – Continuous administration of the nutraceutical (2 mg) along with the antipsychotic olanzapine for an additional 72 h after 72 h of OLZ treatment. Scale bar: 20 μm. (B) Histogram summarizing the mean values for lipid particle count under different experimental conditions. The values were obtained from an analysis using the ImageJ software. Each bar represents the mean ± standard error. Statistical comparison was performed using an unpaired Student’s *t*-test between each treatment group and untreated cells, as well as between the different treatment groups. **p < 0.001; ***p < 0.0001. (C) Histogram summarizing the mean values for the total area occupied by lipid particles under different experimental conditions. The values were obtained from the analysis carried out using the ImageJ software. Each bar represents the mean ± standard error. Statistical comparison was performed using an unpaired Student’s *t*-test between each treatment group and untreated cells, as well as between the different treatment groups. *p < 0.03; ***p < 0.0001.

Exposure of cells to OLZ resulted in a statistically significant increase in lipid droplet count (*p* < 0.0001) and size expressed as area occupied by droplet staining (*p* < 0.0001) (Figure 3).

Student’s *t*-test comparison demonstrated significantly reduced lipid droplet size (*p* < 0.03) and count (*p* < 0.001) in the OLZ + NUTR condition compared to the OLZ-only condition (Figure 3B, C). Importantly, a comparison between the OLZ + NUTR and the UNTR conditions revealed no statistically significant difference in lipid droplet count and size.

The results of the experiments comparing the effects of cell exposure to OLZ + SEMOL versus OLZ are described separately in the Supplementary Materials section.

## Discussion

In the current study, we aimed to probe the beneficial effect of an experimentally designed pasta on non-HDL-C serum levels in patients with MetS, including individuals with comorbid MPDs. We found that individuals consuming experimental pasta had a statistically significant reduction in serum non-HDL-C levels compared to those consuming conventional pasta after 3 months. Several molecular mechanisms may support the observed cholesterol-lowering effect of the experimental pasta we tested. Compared to commonly available SEMOL pasta, experimental pasta was enriched with nutritional elements, such as phytosterols, arabinoxylans, PUFAs, MUFAs, and vitamin E, known to have a beneficial impact on serum cholesterol levels – as reviewed above – and was designed to have a reduced percentage of carbohydrates, notoriously contributing to hypercholesterolemia [87]. Therefore, it is possible that the concurrent and synergistic effects of different compounds contained in experimental pasta contributed to the observed hypocholesterolemic effects of this food.

The experimental pasta achieved an average reduction in non-HDL-C of ~18.5 mg/dl, equivalent to a relative decrease of about 13.5% from baseline levels. Although moderate in magnitude, this reduction is clinically meaningful in the context of cardiovascular disease prevention. In fact, non-HDL-C comprises cholesterol carried by all the atherogenic lipoprotein particles, such as LDL, lipoprotein(a), intermediate-density lipoprotein, and very-LDL remnants, and is strongly predictive of cardiovascular events [8–11]. Consistently, while the European Society of Cardiology recommends a non-HDL-C goal of <100 mg/dl in high-risk patients [7], meta-analytic evidence indicates that each percentage point reduction in non-HDL-C achieved via lipid-lowering therapy corresponds to ~1% relative reduction in coronary heart disease risk [88]. Thus, extrapolating from this evidence, the reduction in non-HDL-C observed in the subjects consuming the experimental pasta is expected to translate into a meaningful decrease in cardiovascular risk if sustained over the long term. Achieved without pharmacological interventions, these results highlight the potential role of dietary modification as either an adjunctive or initial therapeutic strategy for managing dyslipidemia.

Interestingly, our analyses revealed that patients’ polygenic burden for hypercholesterolemia did not interact with the variety of pasta consumed on serum cholesterol reduction, as demonstrated by the absence of a statistical interaction between the TC-PRS and pasta (experimental or placebo) administered in the trial on such a variation. A possible explanation for this result is that the nutrients imparting pro-metabolic properties to the experimental pasta likely influence cholesterol homeostasis, at least in part, by engaging molecular pathways distinct from those harboring genetic variants implicated in the TC-PRS. However, it is important to acknowledge the limitations of PRSs in capturing individual risk, particularly when aiming to develop a clinically predictive tool [89]. Furthermore, future GWAS studies to be conducted on even larger cohorts may yield polygenic scores with greater predictive power than those generated from the GWAS used in this study [28].

Importantly, the findings from the clinical study were corroborated by the *in vitro* experiment conducted on HepG2 cells. These cells, due to their ability to absorb, synthesize, and store lipids, are considered a suitable model for assessing the lipid-modulating effects of nutraceuticals [39, 90]. Notably, this model also recapitulates the lipid dysmetabolism induced by second-generation antipsychotics, particularly OLZ, making it highly relevant for investigating metabolic side effects associated with psychiatric treatments [40, 41, 91–93].

Specifically, OLZ has been demonstrated to induce profound disruptions in lipid homeostasis in HepG2 cells by stimulating de novo lipogenesis – via upregulation of SREBP-1c [92, 94], FASN [95], and ACC [95] – while simultaneously inhibiting fatty acid oxidation through peroxisome proliferator-activated receptor-α downregulation [95]. In addition, OLZ impairs mitochondrial function and triggers endoplasmic reticulum stress, two biological processes contributing to hepatic lipid accumulation [92, 96]. These cellular mechanisms closely mirror the metabolic alterations observed in patients treated with OLZ, supporting the translational relevance of this *in vitro* model. As hypothesized, after observing a marked increase in both the number and size of lipid droplets following exposure to OLZ, in line with previous literature [42, 97], we found that co-administration of the experimental pasta extract alongside OLZ led to a reduction in both the number and size of lipid droplets. This effect may reflect not only a direct modulation of lipid metabolic pathways but also a potential reduction of oxidative stress in hepatocytes. In fact, several of the bioactive components present in the extract, such as vitamin E and polyphenols, are known to mitigate reactive oxygen species accumulation and protect cellular structures from oxidative damage [98–100]. Since OLZ has been shown to induce oxidative stress alongside lipid dysregulation, the observed protective effect of the extract could result from a dual mechanism involving both antioxidant and lipid-modulating actions.

Furthermore, after HepG2 exposure to this combination of compounds, lipid droplet size and number became comparable to those in UNTR cells, indicating that cell exposure to the experimental mixture extract after exposure to OLZ can bring the level of lipid storage in the hepatocyte to pre-exposure conditions.

Notably, our *in vitro* results align with previous evidence showing the effect of natural polyphenolic compounds in inhibiting cholesterol absorption and protecting against oxidative stress, as demonstrated in earlier research using HepG2 cells [101]. However, since lipid droplets in HepG2 cells contain various types of lipids besides cholesterol, the observed reduction may reflect a general effect on lipid accumulation.

This *in vitro* model offered a controlled and translational platform to investigate the direct effects of the bioactive compounds contained in the experimental pasta on hepatocyte lipid metabolism, independently of systemic and behavioral factors that typically affect clinical outcomes. Notably, it effectively recapitulates a key pathogenic mechanism of antipsychotic-induced MetS, namely hepatic lipid accumulation, thereby serving as a bridge between clinical findings and mechanistic understanding [40, 41, 91–93]. The observed reduction in lipid droplet number and size following exposure to the experimental pasta extract suggests a direct effect of the nutraceutical components of this extract on the hepatocyte, possibly mediated by both lipid-modulating and antioxidant mechanisms. These results support the hypothesis that one of the cholesterol-lowering pathways of the experimental pasta involves a reduction in hepatic lipid storage, a critical step in lipid homeostasis and a known target of antipsychotic-induced metabolic disruption [102, 103].

The present findings indicate that integrating a nutritionally enriched pasta into the diet is a feasible and clinically acceptable strategy for lowering atherogenic cholesterol in patients with MetS. This approach leverages a staple food widely consumed in Mediterranean populations, where pasta is an integral component of the habitual diet [104]. Importantly, a recent sensory trial confirmed that the experimental pasta used in the present study and regular pasta have overall comparable palatability [105]. Notably, the reduction in non-HDL-C observed in our trial (~18.5 mg/dl, corresponding to around 13.5%) is comparable to that achieved with established intensive dietary patterns such as the portfolio diet (~14% non-HDL-C reduction) [106] and exceeds cholesterol reductions reported with single dietary interventions like daily phytosterol supplementation (~6–12% LDL-C lowering) [50] or increased soluble fiber intake (~15 mg/dL non-HDL-C reduction) [107]. Nevertheless, further evaluation of the economic feasibility and broader accessibility of this intervention is warranted before widespread clinical implementation.

We are aware of a number of factors that may limit the generalizability of our findings. Notably, there was heterogeneity in the psychopharmacological treatments taken by participants in the group affected by MPD (Supplementary Table 2). However, to reduce the confounding effect of psychotropic treatments, our investigation involved a group of patients with MetS who were not undergoing any psychopharmacological medication. Importantly, our study did not include any intermediate time point between the study baseline and T1, nor any further observation after T1. While potentially reducing the significance of the trial, such a strategy probably minimized dropout rates by reducing the number of clinical evaluations and the time commitment for patients.

Subgroup analyses showed that consumption of the experimental pasta led to reductions in non-HDL-C both in patients with MPD (mean change: −16.682 mg/dl) and those recruited from the INT MED unit (mean change: −19.172 mg/dl). However, the time-by-pasta interaction was significant only in the INT MED subgroup. Interestingly, a reduction in non-HDL-C was observed among patients consuming placebo pasta in the MPD subgroup (mean change: −14.257 mg/dl); therefore, it is possible that such a pronounced placebo effect has contributed to the lack of statistical significance of the time-by-pasta interaction in this subgroup of individuals. In this regard, it is noteworthy that, while significant placebo responses in populations with MPDs are typically reported for psychiatric symptoms [108, 109], these effects have not been systematically investigated for studies with objective metabolic outcomes, such as lipid profiles, in patients with MPDs. Therefore, one plausible explanation for the observed placebo-associated reduction in non-HDL-C might be indirect dietary or lifestyle improvements in the MPD population due to regular participation in the trial, although participants were advised to maintain their usual lifestyle habits. On the other hand, given that lifestyle assessments relied on self-reported questionnaires, such changes, if present, may not have been adequately captured. Additionally, the lack of statistical significance in the MPD subgroup might also reflect limited statistical power resulting from the smaller sample size of this subgroup compared to the INT MED subgroup.

Moreover, it should be noted that the trial was relatively short (3 months), and thus the experimental pasta’s medium- and long-term effects are unknown. Therefore, it is not possible to determine from this study whether the cholesterol reduction would be sustained, magnified, or diminished over time.

Notwithstanding all limitations already highlighted, to our knowledge, this is the first study exploring the hypocholesterolemic effect of an experimental pasta in a mixed population of patients with MetS who developed this disease as a primary condition or as a possible consequence of psychiatric disease and/or related treatments. In this perspective, current results may open the way to future research on the virtuous use of easy-to-obtain ordinary foods modified to improve health and quality of life, especially in individuals with stratified access to good-quality nutrition as a consequence of their social, economic, and clinical status, like patients with MPDs.

## Supporting information

10.1192/j.eurpsy.2025.10057.sm001 D’Ambrosio et al. supplementary material 1 D’Ambrosio et al. supplementary material

10.1192/j.eurpsy.2025.10057.sm002 D’Ambrosio et al. supplementary material 2 D’Ambrosio et al. supplementary material

## Data Availability

The datasets analyzed in this study are not publicly available due to the sensitive nature of the data and the lack of specific informed consent for public data sharing. Access to a limited, de-identified dataset may be granted upon reasonable request to the corresponding author, subject to a data sharing agreement and/or approval from the institutional ethics committee.

## Supplementary material

The supplementary material for this article can be found at http://doi.org/10.1192/j.eurpsy.2025.10057.
