# Supplementary material for: Experimental pasta as an innovative approach to cholesterol reduction in patients with metabolic syndrome, with and without major psychiatric disorders: A randomized controlled trial supported by in vitro validation
Source: Eur Psychiatry. 2025 Jul 7;68(1):e147. doi: 10.1192/j.eurpsy.2025.10057 (PMC12538186; doi:10.1192/j.eurpsy.2025.10057)
Supplement: D’Ambrosio et al. supplementary material 1 — D’Ambrosio et al. supplementary material [file S0924933825100576sup001.docx]

**Supplementary Table 1**

Complete statistical results from the clinical trial.

### A. Primary outcome: non-HDL cholesterol (mg/dL)

| Analysis | Interaction Term | Estimate | SE | t-value | df | 95% CI | p-value |
| --- | --- | --- | --- | --- | --- | --- | --- |
| mITT | time-by-pasta | -10.94 | 3.29 | -3.32 | 251 | [-17.39, -4.49] | 0.001 |
| ITT | time-by-pasta | -9.81 | 3.02 | -3.25 | 277 | [-15.74, -3.89] | 0.001 |
| mITT (adjusted for physical activity) | time-by-pasta | -14.43 | 3.47 | -4.16 | 211 | [-21.24, -7.63] | 0.001 |
| mITT (adjusted for statin therapy) | time-by-pasta | -11.14 | 3.43 | -3.25 | 239 | [-17.87, -4.42] | 0.001 |
| mITT (adjusted for antipsychotic medication) | time-by-pasta | -10.92 | 3.31 | -3.30 | 248 | [-17.41, -4.43] | 0.001 |
| mITT (adjusted for Mediterranean diet adherence score) | time-by-pasta | -12.24 | 3.25 | -3.77 | 241 | [-18.61, -5.88] | 0.001 |
| interaction between time, pasta variety, and TC-PRS | time-by-pasta-by-TC-PRS | 5.48 | 4.13 | 1.33 | 188 | [-2.62, 13.58] | 0.186 |

### B. Exploratory analyses on other lipid parameters

| Outcome | Analysis | Interaction Term | Estimate | SE | t-value | df | 95% CI | p-value |
| --- | --- | --- | --- | --- | --- | --- | --- | --- |
| Total Cholesterol (mg/dL) | mITT | time-by-pasta | -7.95 | 2.82 | -2.81 | 269 | [-13.48, -2.41] | 0.005 |
| Total Cholesterol (mg/dL) | ITT | time-by-pasta | -7.13 | 2.59 | -2.76 | 296 | [-12.21, -2.06] | 0.006 |
| LDL Cholesterol (mg/dL) | mITT | time-by-pasta | -0.01 | 3.13 | 0.00 | 251 | [-6.15, 6.13] | 0.997 |
| HDL Cholesterol (mg/dL) | mITT | time-by-pasta | 2.39 | 1.45 | 1.65 | 268 | [-0.45, 5.23] | 0.101 |
| Triglycerides (mg/dL) | mITT | time-by-pasta | -6.36 | 6.48 | -0.98 | 265 | [-19.07, 6.35] | 0.327 |

### C. Subgroup Analyses. Outcome: non-HDL cholesterol (mg/dL)

| Subgroup | Interaction Term | Estimate | SE | t-value | df | 95% CI | p-value |
| --- | --- | --- | --- | --- | --- | --- | --- |
| INT_MED | time-by-pasta | -14.07 | 3.24 | -4.34 | 183 | [-20.42, -7.72] | < 0.001 |
| MPD | time-by-pasta | -2.42 | 8.34 | -0.29 | 68 | [-18.77, 13.92] | 0.772 |

Abbreviations: SE, standard error; CI, confidence interval; df, degrees of freedom; mITT, modified intention-to-treat; ITT, intention-to-treat; TC-PRS, polygenic risk score for hypercholesterolemia; INT MED, internal medicine sample; MPD, major psychiatric disorders sample.

**Supplementary Table 2**

Psychopharmacological treatment and diagnosis of the subjects who completed the trial

|  |  | **Experimental pasta** | **Conventional pasta** | **Total** |
| --- | --- | --- | --- | --- |
|  |  |  |  |  |
| **Psych. treatment** | Antidepressants (n) | 13 | 17 | 30 |
|  | Antipsychotics (n) | 31 | 22 | 53 |
|  | Mood stabilizers (n) | 10 | 14 | 24 |
|  | Benzodiazepines (n) | 6 | 21 | 27 |
|  |  |  |  |  |
| **Diagnosis** | Schizophrenia (n) | 5 | 4 | 9 |
|  | Schizoaffective Disorder (n) | 4 | 3 | 7 |
|  | Psychosis NOS (n) | 3 | 1 | 4 |
|  | Bipolar Disorder (n) | 9 | 14 | 23 |
|  | Major Depressive Disorder (n) | 1 | 5 | 6 |
|  | Anxiety disorders/OCD (n) | 2 | 2 | 4 |
|  | Other disorders (n) | 12 | 8 | 20 |
