# Supplementary material for: Experimental pasta as an innovative approach to cholesterol reduction in patients with metabolic syndrome, with and without major psychiatric disorders: A randomized controlled trial supported by in vitro validation
Source: Eur Psychiatry. 2025 Jul 7;68(1):e147. doi: 10.1192/j.eurpsy.2025.10057 (PMC12538186; doi:10.1192/j.eurpsy.2025.10057)
Supplement: D’Ambrosio et al. supplementary material 2 — D’Ambrosio et al. supplementary material [file S0924933825100576sup002.docx]

# Supplementary Materials

## Supplementary Methods

### Clinical trial – inclusion and exclusion criteria

Inclusion criteria were age 18-65, meeting the criteria for MetS [1]. Concomitant medications, including anti-diabetic and anti-hypertensive drugs, were allowed based on the requirement that they would be kept stable even in dosage for the duration of the whole trial. Participants were excluded if they met any of the following conditions: pregnant or postpartum women; any difficulty in understanding the proposed questionnaires; the presence of a psychiatric disorder according to DSM-5 or taking psychopharmacological treatment (for the internal medicine group); substance abuse or dependence within six months prior to screening; history of head trauma with loss of consciousness; medical conditions impacting metabolism other than MetS, including Type 1 diabetes mellitus, Cushing's disease, thyroid disorders (hypo- or hyperthyroidism), pheochromocytoma, glucagonoma, neoplastic diseases, severe hepatic insufficiency, severe renal insufficiency, previous bariatric surgery, recent cardiovascular events (myocardial infarction, stroke) within 90 days, Class IV heart failure, alcoholism; intellectual disability; treatment with appetite-reducing drugs or other pharmacological treatments that could influence the participants' dietary regimen.

### Clinical trial – genotyping and PRS calculation

All individuals in the study were genotyped with the Illumina Global Diversity Array v1.0 BeadChip platform. We performed quality control analysis on genotype sequencing data with PLINK v2 [3] following the standard Rapid Imputation and Computational Pipeline (RICOPILI) [4]. Particularly, we retained SNPs with a genotype call rate > 95% and individuals with a sample call rate > 95%. We removed outliers for heterozygosity (F > 0.2) and checked for individuals with non-matching genetic sex with reported sex. We also removed SNPs that were not in Hardy Weinberg Equilibrium (p < 1x10^-6^) and SNPs with a minor allele frequency < 1%. We pre-phased [5] our samples for imputation with SHAPEIT v1.0.1 [6] using the 1000 genomes dataset [7] as a reference for strand alignment, flipping misaligned SNPs based on alternative allele frequency, SNP call rate and χ2 (reference panel vs. data). We used the Michigan Imputation Server [8] to impute variants, setting the parameters to 20 Mb size chunks, and used 1000 Genome Phase 3 v5 [8], as a reference and GRCh37 / hg19 as the array build. Michigan imputation server uses Minimac4 for imputation and Eagle v2.4 [9] for phasing. Finally, we used imputed variants with a maximum genotype imputation probability > 0.9. For principal component analysis, we excluded variants major histocompatibility complex region (Chr6:25-35Mb) and in the chromosome 8 inversion region (Chr8:7-13Mb) to avoid misleading or dubious variants. We performed pruning to minimize linkage disequilibrium between SNPs excluding variants with an R2 below 0.2, in a window of 200 SNPs. We then calculated Eigenvectors and Eigenvalues of the covariance matrix with the R package SNPRelate [10] to identify principal components of the genotype data. For PRS calculation, we used the high-quality imputed genotype data and the weights for each SNP derived from the GWAS on total blood cholesterol [2] for allelic scoring and the 1000 genomes [7] data as a reference. We calculated continuous shrinkage polygenic risk score (PRScs) [11] to include local LD patterns, by incorporating Bayesian modeling to better account for the complex genetic architecture of traits. A more detailed description of PRScs calculation can be found elsewhere [11]. PRS have been adjusted for the first 10 principal components to account for population stratification and scores have been normalized on the population of individuals used in this study. Genotyping data were unavailable for 68 subjects; consequently, TC-PRSs could not be calculated for these individuals.

### In vitro HepG2 experiment - Cell culture and treatments

HepG2 human hepatocarcinoma cell line was cultured in Dulbecco’s Modified Eagle’s Medium (DMEM) High Glucose (EuroClone S.p.a, Milan, Italy) supplemented with 1% penicillin-streptomycin (EuroClone S.p.a, Milan, Italy), 2 mM L-glutamine (Gibco, Thermo Fisher, USA), 10% (vol/vol) Fetal Bovine Serum (FBS) (Gibco, Thermo Fisher, USA). Cells were maintained at 37°C and 5% CO_2_ in a humidified incubator. 2x10^4^ cells were seeded on each glass coverslip. Cells were left to attach for 24 h and then exposed for the first 72 hours to olanzapine (OLZ) 10 µM and for the next 72 hours, either to the extract derived from the experimental mixture (NUTR) or to semolina (SEMOL) (2 mg). The components of the experimental mixture and semolina were provided by Casillo Next Gen Food srl (Corato, BA, Italy). In order to control for any biasing effect induced by solvents used to dissolve olanzapine and experimental pasta extract on HepG2 lipid droplet count, exposure to these solvents, namely dimethyl sulfoxide (DMSO) and ethanol, was used as a control condition in experiments on HepG2 cell lines (data not shown).

### Experimental mixture and semolina extract

Semolina extract was obtained by dissolving 100% semolina in 70% ethanol. The experimental mixture extract was prepared by mixing 27% de-oiled wheat germ, 67% refined semolina, and 6% durum wheat oil, reflecting the proportions adopted for the experimental pasta production and resuspending the mixture in 70% ethanol. Ethanol was selected for its efficiency in extracting alcohol-soluble bioactive compounds, including polyphenols, phytosterols, polyunsaturated fatty acids, and vitamin E, which are naturally present in wheat germ and bran and are known to exert lipid-modulating and antioxidant effects in hepatic cells [12–14]. After an overnight stirring, the insoluble fraction settled, and the supernatant was collected in a centrifuge tube. The supernatant was dehydrated and then reconstituted in phosphate-buffered saline solution at a concentration of 100 mg/ml.

### Lipid droplet staining and imaging analysis

HepG2 cells, grown on coverglass slides under different experimental conditions, were fixed with 3.7% paraformaldehyde for 30 minutes. After being washed twice with distilled water, the cells were treated with 60% isopropanol (PanReac AppliChem) for 5 minutes before staining with 0.5% Oil Red O (Sigma Aldrich, St. Louis, MO, USA) for 20 minutes at room temperature. The Oil Red O solution was then discarded, and the cells were washed 2–5 times with water until no excess stain was visible. The cells were then observed under a Leica Microsystems DMi8 microscope (Leica Microsystems Inc., Wetzlar, Germany) at 63x magnification in brightfield. A technical triplicate was carried out for each tested condition. For each sample, images of four fields were acquired and processed. The experiment was replicated three times, resulting in a total of 36 image fields for each experimental condition. ImageJ software and the methodological protocol provided by Adomshick et al. [15] were used to analyze the images (Figure 3A) and quantify lipid particles as well as the total area occupied by them in the brightfield microscopy images.

## Effects of HepG2 exposure to the combination of olanzapine and semolina.

In order to assess the impact of the semolina portion, which represents the main component of both conventional and experimental pasta in our trial, on lipid accumulation in the HepG2 cells, we exposed cells to OLZ, SEMOL, OLZ + SEMOL, and to vehicle (UNTR) and compared cell lipid droplet size and number across these conditions.

Student’s t-test was used for inter-condition comparison, and statistical significance was set for p-value < 0.05.

Comparison between the OLZ and OLZ+SEMOL conditions revealed that the size, but not the count of the lipid droplets was significantly reduced in the OLZ+SEMOL condition compared to OLZ alone (p<0.01) (Supplementary Figure 1), suggesting that semolina could have a lipid-lowering potential *per se*. Although there is currently no direct evidence regarding the effects of semolina extract on cells, certain observations indicate that durum wheat, the source of semolina, is rich in polyphenols and antioxidant compounds. These substances are known to activate molecular pathways within cells, crucial for promoting lipid oxidation and reducing lipid synthesis, which could facilitate the reduction of lipid accumulation [16,17]. However, unlike in the comparison between the OLZ+NUTR and UNTR conditions, lipid droplet number and area in cells exposed to OLZ+SEMOL were significantly higher than in untreated (UNTR) cells (p<0.01 and p<0.001, respectively), likely indicating that semolina has a weaker potential than the experimental mixture extract to mitigate olanzapine-induced hepatocyte lipid accumulation. In line with this hypothesis, when comparing the count of lipid droplets in cells exposed to OLZ+SEMOL and in those exposed to OLZ+NUTR, we observed that the lipid droplet number in the OLZ+SEMOL condition was significantly higher (p<0.0001) than in the OLZ+NUTR condition.

# Supplementary Figure Legends

Supplementary Figure 1

**Effect of Semolina and Olanzapine Treatments on Lipid Droplet Formation in HepG2 Cells.**

The conditions are as follows: SEMOL: treatment with a 2 mg semolina extract for 72 hours; OLZ: treatment with 10 µM olanzapine for 72 hours; OLZ+SEMOL: Continuous administration of semolina (2 mg) along with the antipsychotic olanzapine for an additional 72 hours after 72 hours of OLZ treatment.

**A.** Histogram summarizing the mean values ​​for the lipid particle count under different experimental conditions, obtained from the analysis carried out using the ImageJ software. Each bar represents the mean±standard error. Statistical comparison was performed using unpaired Student’s *t* test between each treatment group and untreated cells, as well as between the different treatment groups **p*<0.01; ***p*<0.001

**B.** Histogram summarizing the mean values ​​for the total area occupied by lipid particles in the different experimental conditions, obtained from the analysis carried out using the ImageJ software. Each bar represents the mean±standard error. Statistical comparison was performed using unpaired Student’s *t* test between each treatment group and untreated cells, as well as between the different treatment groups **p*<0.01; ** *p*< 0.001; ****p*<0.0001

# Supplementary Table Legends

Supplementary Table 1

**Complete statistical results from the clinical trial**

Supplementary Table 2

# Psychopharmacological treatment and diagnosis of the subjects who completed the trial

# Bibliography

[1] Alberti KGMM, Zimmet P, Shaw J. Metabolic syndrome—a new world‐wide definition. A Consensus Statement from the International Diabetes Federation. Diabet Med 2006;23:469–80. <https://doi.org/10.1111/j.1464-5491.2006.01858.x>.

[2] Graham SE, Clarke SL, Wu K-HH, Kanoni S, Zajac GJM, Ramdas S, et al. The power of genetic diversity in genome-wide association studies of lipids. Nature 2021;600:675–9. <https://doi.org/10.1038/s41586-021-04064-3>.

[3] Purcell S, Neale B, Todd-Brown K, Thomas L, Ferreira MAR, Bender D, et al. PLINK: A Tool Set for Whole-Genome Association and Population-Based Linkage Analyses. Am J Hum Genet 2007;81:559–75. <https://doi.org/10.1086/519795>.

[4] Lam M, Awasthi S, Watson HJ, Goldstein J, Panagiotaropoulou G, Trubetskoy V, et al. RICOPILI: Rapid Imputation for COnsortias PIpeLIne. Bioinformatics 2020;36:930–3. <https://doi.org/10.1093/bioinformatics/btz633>.

[5] Howie B, Fuchsberger C, Stephens M, Marchini J, Abecasis GR. Fast and accurate genotype imputation in genome-wide association studies through pre-phasing. Nat Genet 2012;44:955–9. <https://doi.org/10.1038/ng.2354>.

[6] Delaneau O, Marchini J, Zagury J-F. A linear complexity phasing method for thousands of genomes. Nat Methods 2012;9:179–81. <https://doi.org/10.1038/nmeth.1785>.

[7] Clarke L, Fairley S, Zheng-Bradley X, Streeter I, Perry E, Lowy E, et al. The international Genome sample resource (IGSR): A worldwide collection of genome variation incorporating the 1000 Genomes Project data. Nucleic Acids Res 2017;45:D854–9. <https://doi.org/10.1093/nar/gkw829>.

[8] Das S, Forer L, Schönherr S, Sidore C, Locke AE, Kwong A, et al. Next-generation genotype imputation service and methods. Nat Genet 2016;48:1284–7. <https://doi.org/10.1038/ng.3656>.

[9] Loh P-R, Danecek P, Palamara PF, Fuchsberger C, Reshef YA, Finucane HK, et al. Reference-based phasing using the Haplotype Reference Consortium panel. Nat Genet 2016;48:1443–8. <https://doi.org/10.1038/ng.3679>.

[10] Zheng X, Levine D, Shen J, Gogarten SM, Laurie C, Weir BS. A high-performance computing toolset for relatedness and principal component analysis of SNP data. Bioinformatics 2012;28:3326–8. <https://doi.org/10.1093/bioinformatics/bts606>.

[11] Ge T, Chen C-Y, Ni Y, Feng Y-CA, Smoller JW. Polygenic prediction via Bayesian regression and continuous shrinkage priors. Nat Commun 2019;10:1776. <https://doi.org/10.1038/s41467-019-09718-5>.

[12] Assis AM de, Rech A, Longoni A, Rotta LN, Denardin CC, Pasquali MA, et al. Ω3-Polyunsaturated fatty acids prevent lipoperoxidation, modulate antioxidant enzymes, and reduce lipid content but do not alter glycogen metabolism in the livers of diabetic rats fed on a high fat thermolyzed diet. Mol Cell Biochem 2012;361:151–60. <https://doi.org/10.1007/s11010-011-1099-4>.

[13] Jian L, Xue Y, Gao Y, Wang B, Qu Y, Li S, et al. Vitamin E Can Ameliorate Oxidative Damage of Ovine Hepatocytes In Vitro by Regulating Genes Expression Associated with Apoptosis and Pyroptosis, but Not Ferroptosis. Molecules 2021;26:4520. <https://doi.org/10.3390/molecules26154520>.

[14] Machado IF, Miranda RG, Dorta DJ, Rolo AP, Palmeira CM. Targeting Oxidative Stress with Polyphenols to Fight Liver Diseases. Antioxidants 2023;12:1212. <https://doi.org/10.3390/antiox12061212>.

[15] Adomshick V, Pu Y, Veiga-Lopez A. Automated lipid droplet quantification system for phenotypic analysis of adipocytes using CellProfiler. Toxicol Mech Methods 2020;30:378–87. <https://doi.org/10.1080/15376516.2020.1747124>.

[16] Dzah CS, Asante-Donyinah D, Letsyo E, Dzikunoo J, Adams ZS. Dietary Polyphenols and Obesity: A Review of Polyphenol Effects on Lipid and Glucose Metabolism, Mitochondrial Homeostasis, and Starch Digestibility and Absorption. Plant Foods Hum Nutr 2023;78:1–12. <https://doi.org/10.1007/s11130-022-01034-6>.

[17] He L, Su Z, Wang S. The anti-obesity effects of polyphenols: a comprehensive review of molecular mechanisms and signal pathways in regulating adipocytes. Front Nutr 2024;11:1393575. <https://doi.org/10.3389/fnut.2024.1393575>.
